# Pancreatic acinar cell carcinoma—case report and literature review

**DOI:** 10.1186/s12885-018-5008-z

**Published:** 2018-11-08

**Authors:** Zhang Xing-mao, Zhang Hong-juan, Li Qing, He Qiang

**Affiliations:** 1 Department of hepatobiliary surgery, Beijing Chaoyang Hospital, Capital Medical University, 8 Gongti South Street, Chaoyang District, 100021 Beijing China; 2 Department of general surgery, The 2nd Hospital of Chengde Medical College, Chengde Central Hospital, Chengde, Hebei province China; 3 Department of pathology, Beijing Chaoyang Hospital, Capital Medical University, Beijing, China

**Keywords:** Acinar cell carcinoma, Pancreas, Diagnosis, Treatment, Prognosis

## Abstract

**Background:**

Pancreatic acinar cell carcinoma (ACC) is a rare tumor that constitutes 1% of all pancreatic neoplasms. Pancreatic ACC has unique characteristics in terms of biological behavior, imaging and prognosis.

**Case presentation:**

The present study reported two cases of pancreatic ACC confirmed by postoperative pathology and both cases exhibited several different imaging features and laboratory test results. Both cases had approximately 4 cm mass located in uncinate process of pancreas. Dilated intra- and extra-hepatic bile ducts was observed in one case, along with calcification. Heterogeneous enhancement of the tumor was exhibited in both patients with different intensities. Obstructive jaundice, elevated α-fetoprotein and CA 19–9 was found in one case, while the other case had normal liver function and tumor markers.

**Conclusions:**

It was difficult to accurately diagnose pancreatic ACC before the operation despite its unique characteristics. Radical resection was the best treatment modality for resectable pancreatic ACC.

## Background

Accounting for only 1% of all pancreatic tumors, pancreatic acinar cell carcinoma (ACC), which originates from acinar elements of the exocrine pancreas, is a rare neoplasm [1, 2]. Pancreatic ACC has been better understood since the first report by Berner in 1908 [3]. Pancreatic ACC has unique characteristics in terms of biological behavior, imaging and prognosis relative to pancreatic ductal adenocarcinoma, such as elevated α-fetoprotein (AFP) level in some patients [4, 5], relatively longer survival [6, 7], etc. Suspected diagnosis of pancreatic ACC in patients who are fit for operation mainly relies on imaging examinations including enhanced computed tomography (CT) or magnetic resonance imaging (MRI), and confirmed diagnosis depends on postoperative pathology. Herein, we described two cases with pathologically confirmed pancreatic ACC, who presented with different manifestations of the same disease.

## Case presentation

### Case one

A 69-year-old male patient was admitted to our hospital with the chief complaint of jaundice of skin and sclera accompanied by epigastric pain for two weeks. Further examinations including enhanced abdominal and pelvic CT scans, chest X-ray, abdominal ultrasound, tumor markers, liver and renal function and coagulation function were performed.

**Fig. 1:**
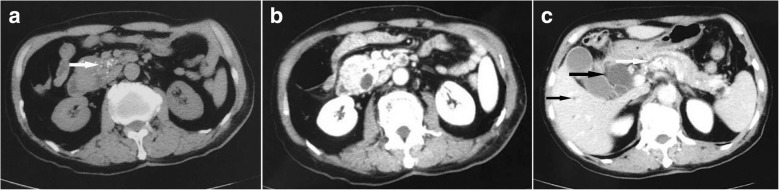
**a**. A mass of approximately 4.0 cm diameter located in uncinate process of pancreas, the white arrow shows the calcification in the mass; **b**. The tumor was significantly enhanced in the arterial phase, which was similar to the surrounding pancreatic parenchyma; **c**. The black arrow shows that the intra- and extra-hepatic bile ducts were obviously dilated and the white arrow shows the slightly dilated pancreatic duct

CT revealed a low-density mass of 4.0 cm diameter located in uncinate process of pancreas, obviously dilated intra- and extra-hepatic bile ducts and slightly dilated pancreatic duct. Non-contrast CT scan showed calcification in the mass. Contrast CT showed that enhancement of the tumor was similar to surrounding normal pancreatic parenchyma (Fig. 1). The laboratory data were as follows: white blood cell (WBC) count, 4.6 × 10^9^/L (normal: 4.0–10.0 × 10^9^/L); red blood cell (RBC) count, 4.3 × 10^12^/L (normal: 3.5–5.5 × 10^12^/L); hemoglobin (Hgb), 125 g/L (normal: 120–160 g/L); AFP, 71.5 ng/mL (normal: < 8.1 ng/mL); carcinoembryonic antigen (CEA), 2.0 ng/mL (normal: 0–5.0 ng/mL); carbohydrate antigen 19–9 (CA 19–9), 437.2 U/mL (normal: 0–37 U/mL); aspartate transaminase (AST), 51 U/L (normal: 15–40 U/L); alanine transaminase (ALT), 151 U/L (normal: 9–50 U/L); total bilirubin (TBIL), 281.2 μmol/L (normal: 5.0–21.0 μmol/L); direct bilirubin (DBIL), 212.6 μmol/L (normal: 0–6.8 μmol/L).

Based on these results, an incorrect diagnosis of pancreatic neuroendocrine neoplasm was suspected before the operation and pancreaticoduodenectomy was performed on this patient. Pancreatic ACC with invasion of duodenum and distal common bile was confirmed by postoperative pathology, and no metastatic lymph nodes were found. Gemcitabine-based regime was administered to this patient one month after the operation. The patient was followed-up with physical examination, laboratory tests, and imaging examinations every three months and was alive without relapse at nine months after the operation.

### Case two

A 79-year-old male patient, without any clinical symptoms, was found to have a pancreatic mass by ultrasound during routine physical examination. After he was admitted to our center, we also performed further examinations including enhanced abdominal and pelvic CT scans, chest X-ray, tumor markers, liver and renal function, coagulation function, etc.

**Fig. 2:**
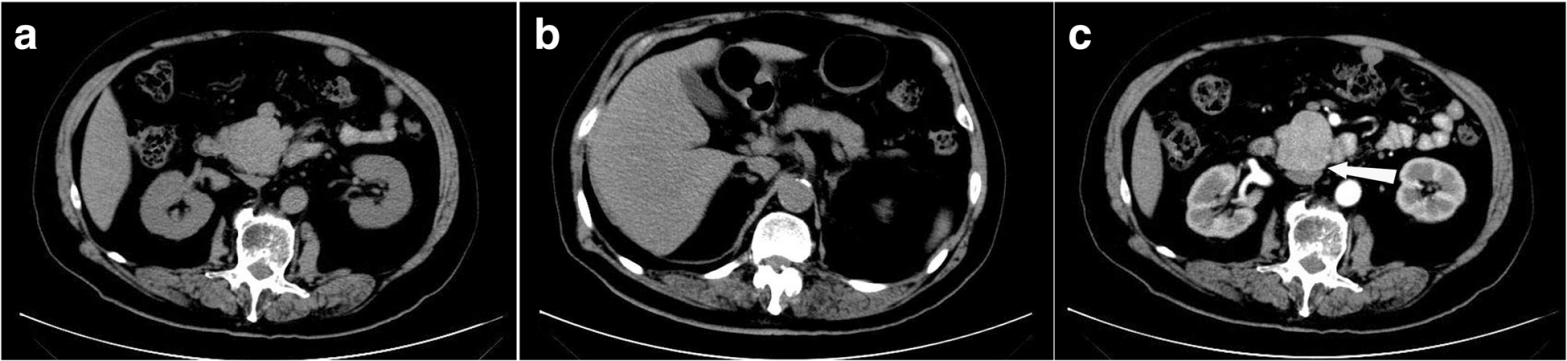
**a**. An irregular mass with well-defined tumor margin located in uncinate process of pancreas; **b**. No dilated intra- and extra-hepatic bile ducts or pancreatic duct was found; **c**. The white arrow shows the enhanced capsule of the tumor

The CT images showed an irregular mass with the greatest diameter of about 4.5 cm located in uncinate process of pancreas, with well-defined margins. No dilated intra- and extra-hepatic bile ducts were found, and pancreatic duct was normal. In the arterial phase, heterogeneous enhancement of the tumor was seen, which was less intense than the normal surrounding pancreatic parenchyma, and enhanced capsule was found (Fig. 2). The laboratory data were as follows (normal ranges were the same as above): WBC count, 6.9 × 10^9^/L; RBC count, 4.6 × 10^12^/L; Hgb, 151 g/L; AFP, 4.0 ng/mL; CEA, 1.49 ng/mL; CA 19–9, 14.2 U/mL; AST, 57 U/L; ALT, 73 U/L; TBIL, 11.5 μmol/L; and DBIL, 4.4 μmol/L.

Pancreatic ACC was suspected before the operation and pancreaticoduodenectomy was performed on this patient. Pancreatic ACC was confirmed by postoperative pathology, with no metastatic lymph nodes. The patient rejected chemotherapy and routine follow-up was conducted. No recurrence was found one year after the operation.

## Discussion and conclusions

Acinar cell carcinoma (ACC) represents approximately 1% of all pancreatic neoplasms, which primarily occur in late adulthood [8, 9], with a male to female ratio of 3.6:1 [10]. Most patients with pancreatic ACC have no specific symptoms, and the non-specific clinical symptoms include weight loss (52%), abdominal pain (32%), nausea and vomiting (20%), melena (12%), weakness, anorexia or diarrhea (8%) [11].

Pancreatic ACC is often misdiagnosed as pancreatic duct adenocarcinoma or pancreatic neuroendocrine tumor although it has unique characteristics in terms of radiological findings, laboratory examinations, etc. Pancreatic ACC typically has a large size when detected, with a diameter > 10 cm [11], and lesions with a diameter < 2 cm are rarely detected. In radiological images, pancreatic ACC usually appears well marginated, with a thin, enhanced capsule in approximately 60% of patients, and central hypodensity and calcification are common. Unlike pancreatic duct adenocarcinoma, which typically has ductal obstruction due to its origin in intraductal epithelial cells, ductal obstruction may be either mild or absent in ACC located in the pancreatic head [12]. This characteristic is used to differentiate from pancreatic duct adenocarcinoma, but it is not a specific feature of this tumor. Tumor exhibits hypodensity in plain scan, and mild to moderate heterogeneous enhancement in arterial phase. In most cases, enhancement of tumor is less intense than the surrounding normal pancreatic parenchyma. However, the enhancement of tumor was similar to the surrounding parenchyma in case one in this study.

Due to the unique ability to produce pancreatic enzymes, approximately 10–15% of patients develop lipase hypersecretion syndrome, a type of paraneoplastic syndrome with multiple nodular foci of subcutaneous fat necrosis and polyarthralgia [13]. Although this syndrome could occasionally occur due to an extremely large organ-limited primary carcinoma, it is more commonly encountered in patients with hepatic metastasis. Patients with lipase hypersecretion syndrome were found to have a particularly short survival [10]. Serum lipase can decrease to normal level after successful surgical removal of the tumor, which resolves the lipase hypersecretion syndrome. Serum tumor markers are not consistently elevated in patients with pancreatic ACC, but increased serum alpha-fetoprotein level can be found in some patients.

The diagnosis of pancreatic ACC can be preoperatively confirmed by biopsy, such as fine needle aspiration, but tumor cells may occasionally be difficult to identify with fine needle aspiration alone due to the highly cellular nodules of monotonous tumor cells with little or no stroma and the lack of a desmoplastic response [11].

The best therapeutic regimen is comprehensive treatment based on radical resection [14]. Patients with resectable lesion can benefit from surgical removal. Holen et al. [11] reported that patients with pancreatic ACC who received radical resection had a median survival of 36 months, as compared to only 14 months for patients without surgery. Wang et al. [3] showed that patients who received resection had a median survival of 19 months, but patients without operation had a significantly shorter survival, with a median of only nine months. There is no consensus on adjuvant therapy for resected pancreatic ACC. Some studies suggested that patients could benefit from 5-FU based or gemcitabine-based chemotherapy after the resection of pancreatic ACC [15–17]. There is no standard chemotherapy regime for unresectable pancreatic ACC cases. Yoo et al. [18] confirmed that oxaliplatin-based chemotherapy had improved activity against pancreatic ACC as compared to gemcitabine. Hashimoto et al. [9] suggested that modified FOLFIRINOX was safe and effective in the treatment of pancreatic ACC.

In summary, pancreatic ACC, as a rare neoplasm, has different manifestations. Surgical resection is the first choice for a resectable lesion regardless of tumor size. There is no consensus on adjuvant therapy.

## Abbreviations

ACC: Acinar cell carcinoma
AFP: α-fetoprotein
ALT: Alanine transaminase
AST: Aspartate transaminase
CA 19–9: Carbohydrate antigen 19–9
CEA: Carcinoembryonic antigen
CT: Computed tomography
DBIL: Direct bilirubin
FOLFIRINOX: leucovorin and fluorouracil plus irinotecan and oxaliplatin
Hgb: Hemoglobin
MRI: Magnetic resonance imaging
RBC: Red blood cell
TBIL: Total bilirubin
WBC: White blood cell
